# An Application of a Service-oriented System to Support ArrayAnnotation in Custom Chip Design for Epigenomic Analysis

**Published:** 2008-04-10

**Authors:** Junghee Han, Dustin Potter, Tahsin Kurc, Greg Singer, Pearlly S. Yan, Sun Hao, Shannon Hastings, Stephen Langella, Scott Oster, Ramana V. Davuluri, Tim H.-M. Huang, Joel H. Saltz

**Affiliations:** Department of Biomedical Informatics and Department of Molecular Virology, Immunology, and Medical Genetics The Ohio State University Columbus, OH, U.S.A

**Keywords:** service oriented architectures, grid computing, caBIG and caGrid, data integration, epigenetic studies

## Abstract

We present the implementation of an application using caGrid, which is the service-oriented Grid software infrastructure of the NCI cancer Biomedical Informatics Grid (caBIG^TM^), to support design and analysis of custom microarray experiments in the study of epigenetic alterations in cancer. The design and execution of these experiments requires synthesis of information from multiple data types and datasets. In our implementation, each data source is implemented as a caGrid Data Service, and analytical resources are wrapped as caGrid Analytical Services. This service-based implementation has several advantages. A backend resource can be modified or upgraded, without needing to change other components in the application. A remote resource can be added easily, since resources are not required to be collected in a centralized infrastructure.

## 1. Introduction

The development of high-throughput technologies has provided powerful tools for comprehensive analysis of gene expression, chromosomal alterations, and nucleotide mutations in cancer genomes. This enables a new paradigm for today’s research of complex cancer genomes. The outcomes are potentially enormous for the improvement of cancer diagnosis and prognosis. Until recently, such a high-throughput technological development was lacking for the emerging phenomenon of epigenetic alterations in cancer. Unlike genetic mutations, these molecular changes do not affect DNA sequences and yet can stably influence gene expression during cancer development. These modifications, including DNA methylation in GC-rich CpG islands as well as methylation and acetylation of histone lysines, are responsible for altered chromatin structure that affects gene expression patterns during cancer initiation and development (Baylin and Ohm, 2006; Yoo and Jones, 2006). Recently, novel epigenomic microarray platforms have been developed (Yan, Chen et al. 2001; Weinmann, Yan et al. 2002) and used to identify molecular subtypes of breast (Yan, Chen et al. 2001), ovarian (Wei, Chen et al. 2002), colorectal tumors (Yan, Efferth et al. 2002), leukemia (van Doorn, Zoutman et al. 2005) and gliomas (Waha, Guntner et al. 2005).

Studies on epigenetic alterations in cancer generate and reference vast amounts of epigenomic data. They require integration and processing of information from different datasets and data types for both the design of microarray experiments and the analysis of experimental results. Different types of experiments for gene expression, DNA methylation, and chromatin modifications are carried out in epigenetic studies. Differential methylation hybridization (DMH) (Yan, Chen et al. 2001) experiments, for example, determine the methylation status of promoter CpG islands in cancer genomes. Chromatin immunoprecipitation microarray (also called ChIP-chip) (Weinmann, Yan et al. 2002) is used to identify profiles of chromatin remodeling and patterns of differential binding of transcription factors in cancer cells. The results from these experiments are analyzed to examine the relationship of epigenomic alterations to clinicopathological features. The complexity and heterogeneity of data sets and analysis operations makes it difficult for researchers to manage and process information in their studies. It is also desirable for collaborating researchers to be able to share their data and analysis tools.

This paper describes the use of the caGrid infrastructure (Saltz, Oster et al. 2006) in developing an application, referred to here as ArrayAnnotator-G, to support array annotation for interrogation of cancer epigenomes. Array annotation is an important operation for both experiment design as well as analysis of the results. Table 1 shows several types of array annotations that can affect analysis and/or the design of an experiment. For example, computing annotation information for target genes of interest is critical to accurately normalize and analyze DMH experimental data. Knowing the genomic location of 60-mer oligonucleotides in a given microarray—whether the oligonucleotides are within a gene’s promoter region or not—can help researchers to interpret their experiments, because it is known that the methylation of a gene’s promoter region affects transcription of the gene. caGrid is the service-based Grid software infrastructure of the cancer Biomedical Informatics Grid (caBIG^TM^) program (https://cabig.nci.nih.gov). It provides tools, application programming interfaces, and runtime environment that enable an application developer to stand up secure data and analytical services and to implement client programs which can access multiple services hosted locally or remotely at other institutions. In the current implementation of ArrayAnnotator-G presented in Section 4, we use DMH experiments as an example to explain the implementation of the system. The same principles can be applied to support ChIP-chip experiments.

The caGrid-based implementation of ArrayAnnotator-G allows for the separation of the implementation of backend resources from their interface to the environment. Each data source in ArrayAnnotator-G is implemented as a caGrid Data Service, and analytical resources are wrapped as caGrid Analytical Services. This has several advantages. The implementation of a backend resource can be modified, or the resource can be upgraded, without affecting client programs and other resources in the environment. New and remote resources (e.g. another oligonucleotide information source) can be added easily, since the system does not require that the information and analytical functionality be collected in a centralized infrastructure. The service-based implementation makes it easier to integrate ArrayAnnotator-G in a larger data collection and analysis workflow. Moreover, because it is built on a flexible and scalable Grid-based platform, ArrayAnnotator-G promotes interchange and inter-analysis of a vast amount of heterogeneous data across different research centers as well as within the same institution so that this study helps researchers to better appreciate the underlying cancer biology in overall.

The rest of the paper is organized as follows. In Section 2 we provide a brief overview of Grid computing technologies and the caGrid infrastructure. Section 3 describes the design objectives of the ArrayAnnotator-G application. We have designed ArrayAnnotator-G by modeling epigenetic cancer research carried out at the OSU Center for Integrative Cancer Biology. The three use cases that have motivated the design as well as the application framework to address these use cases are presented in this section. The details of the implementation of the application are described in Section 4. This section presents how the caGrid infrastructure and the tools developed in caBIG^TM^ are used to develop data models for annotation datasets and implement the analytical and data services and client programs in the application. We conclude in Section 5.

## 2. Grid Computing and caGrid

Grid computing started as a mechanism to enable access to high-end computing facilities across multiple supercomputer centers to solve complex scientific and engineering problems that require high computing power (Foster and Kesselman, 1999; Berman, Hey et al. 2003). It has since evolved into a platform that facilitates the development, sharing, and integration of data and analytical resources and to support applications that make use of those resources (Chervenak, Foster et al. 2000; Natrajan, Humphrey et al. 2001; Foster, Kesselman et al. 2002; Foster, Kesselman et al. 2002; Atkinson, Dialani et al. 2003; Foster, 2006; Saltz, Oster et al. 2006). Tools and middleware systems have been developed by the Grid computing community to support a wide range of application requirements, including efficient transfer of large datasets (Thain, Basney et al. 2001; Chervenak, Deelman et al. 2003; Allcock, Foster et al. 2004), implementation and execution of workflows (Beynon, Kurc et al. 2001; Altintas, Bhagwanani et al. 2003; Deelman, Blythe et al. 2003), management of caches and replicas (Andrade, Kurc et al. 2002; [Bibr b10-cin-6-0111][Bibr b34-cin-6-0111]), Grid-enablement of databases (Smith, Gounaris et al. 2002; Hastings, Langella et al. 2004; Antonioletti, Atkinson et al. 2005), metadata services (Deelman, Singh et al. 2004), and security (Foster, Kesselman et al. 1998; Welch, Siebenlist et al. 2003; Bhatia, Chandra et al. 2005; Lang, Foster et al. 2006; Langella, Oster et al. 2006). Grid technologies have been successfully applied in different application domains and projects as well (Peltier and Ellisman, 2003; Solomonides, McClatchey et al. 2003; Tweed and Miguet, 2003; Parashar, Klie et al. 2004; Rogulin, Estrella et al. 2004; Grethe, Baru et al. 2005; Siegel, Siddiqui et al. 2005).

Interoperability is a concern in environments where data and analytical resources are heterogeneous, and when programmatic access to these resources is required. Grid Services standards, namely the Open Grid Services Architecture (OGSA) standards (Foster, Kessellman et al. 2001; Foster, Kesselman et al. 2002; Foster, Kesselman et al. 2002), have been developed through a community effort, overseen by the Global Grid Forum (http://www.ggf.org), to enable interoperability among Grid enabled applications. These standards adapt and extend Web Services for scientific applications. They define mechanisms and guidelines for such additional features as stateful services, service notification, and management of service lifetime, within the context of established Web Services standards. The OGSA has recently evolved into the Web Services Resource Framework (WSRF) (Czajkowski, Ferguson et al. 2004; Foster, Czajkowski et al. 2005). The WSRF extends the core concepts underlying the OGSA and adds new functionality to establish a path towards unifying Web services and Grid services technologies.

The caGrid infrastructure (Saltz, Oster et al. 2006) is the core Grid software architecture of the cancer Biomedical Informatics Grid (caBIG^TM^) effort. caBIG^TM^ is a national-scale program sponsored by the National Cancer Institute to provide solutions to the information management, integration, analysis, and sharing needs of basic and clinical cancer research projects (https://cabig.nci.nih.gov). The caGrid infrastructure provides runtime support and tools for development and deployment of applications in the caBIG environment. It is a services-oriented architecture and builds on the OGSA/WSRF-based Grid Services paradigm^1^. A distinguishing feature of caGrid over the basic OGSA/WSRF based systems is the support for syntactic and semantic interoperability between resources. caGrid is based on a model-driven architecture approach, in which data types served in the environment are curated and semantically harmonized to enable interoperability through object-oriented abstractions, common data elements, and controlled vocabularies. That is, the client and service APIs of the caGrid service are object-oriented and operate on well-defined data objects, which are built from common data elements and controlled vocabularies registered in the caBIG environment. In caGrid, each data or analytical resource is wrapped as a Grid service, exposed through an object oriented view, and accessed through standard communication and service invocation protocols as defined by the OGSA/WSRF standards. A backend data source, for example, may be a relational database; the caGrid Data Service wrapper for the data source exports the data source’s data elements as objects to the environment. Similarly, an analytical resource (e.g. an analysis program) implemented as a caGrid Analytical Service provides methods that take objects as input and return objects as output. Any interaction between two Grid end-points (e.g. between clients and services, and between services) is carried out through Grid communication and service invocation mechanisms and protocols. In addition to resource specific services, caGrid has common coordination services. These services provide the Grid-wide functions required by clients and local services. They include metadata management services, services for advertisement and discovery, query services, and services for security. For instance, the advertisement and discovery services can be used for advertising the availability, functions, and state of services to the caBIG environment and for discovering this information. The coordination services can be replicated and distributed in the environment to achieve higher availability and performance.

caGrid is an open-source, publicly available infrastructure. Researchers can freely download the caGrid infrastructure and its components from the project web site (https://cabig.nci.nih.gov/workspaces/Architecture/caGrid) to use in their projects. Researchers can also request accounts to access the production installation of caGrid maintained by the NCICB. Although the architecture of caGrid is driven mainly by use cases in cancer research, its architecture and implementation is generic to be employed in other biomedical research domains. Indeed, caGrid is being used as the underlying Grid middleware system to build the Cardiovascular Research Grid (http://cvrgrid.org/).

## 3. Design Objectives and Application Framework

Our main design objectives are to develop a software application that is flexible and extensible and that would enable integration and processing of information from decentralized databases. Three interrelated usage scenarios have driven the design of ArrayAnnotator-G: 1) development of an oligonucleotide library; 2) selection of appropriate oligonucleotides for CpG island microarray design, given one or more oligonucleotide libraries/ sources; and 3) annotation of a pre-built microarray in order to support further analysis. In epigenetic studies at our center, this analysis has mostly involved correcting microarray hybridization data by systematically anticipating the effects of binding artifacts.

### Use case 1: development of oligonucleotide library

In this scenario, the goal is to construct an oligonucleotide library that can be accessed by researchers across different institutes and/or by collaborative peers within a single center. This library maintains information for each oligonucleotide so that it can help researchers to design custom arrays for their studies. To build this library, a set of oligonucleotides are selected either from the entire genome or from a list of oligonucleotides provided by microarray chip companies. Annotation information for each oligonucleotide can be obtained by 1) examining and applying analysis methods on its genomic sequence and 2) surveying existing literatures and/or previous studies conducted at the same or other research centers. The sequence information is then annotated to generate annotation information (e.g. GC percentage) for each oligonucleotide. The oligonuleotides with rich annotation information is stored so that they can be shared with other researchers to design arrays. In the example of the application presented in Section 4, we tentatively maintain about 600,000 oligonucleotides. Currently, each entry in this library contains promoter region information, a list of CpG islands near or surrounding the oligonucleotides, GC contents, a gene identification to which the oligonucleotides belong to, Tm value, uniqueness, repeat-masked region, and enzyme cleavage information. This library, of course, can be expanded to incorporate additional DMH experiments and analysis.

### Use case 2: selection of appropriate oligonucleotides for an epigenomic CpG island microarray platform

Within a set of oligonucleotides, a screening process should be performed to select an appropriate subset of oligonucleotides for experiments. To design a custom array, the general features of each oligonucleotide should be examined and understood in addition to its sequence and gene information. For instance, the percentage of GC content and overlap with CpG island regions for each oligonucleotide are important criteria. In addition, researchers might want to computationally predict the effects of their experimental protocols on each oligonucleotide; the actual enzyme cleavage locations of selected restriction enzyme sets (i.e. *Bfa*1., *McrBC*, and *Hin*II) in the DMH experiments would be helpful for researchers to evaluate candidate oligonucleotides and to predict or analyze the experimental results. Thus, a subset of oligonucleotides can be chosen based on the oligonucleotide and annotation information, such as the length of the oligonucleotides, their locations relative to known or hypothetical genes, promoters, or CpG islands regions, and to a methylation-sensitive restriction site (e.g. *Hpa*II, *Hinp*1I, and *Acy*I). In addition, control oligonucleotides can be chosen from a given set of oligonucleotides for quality control purposes. The set of control oligonucleotides contains sequences (1) with little or no match when BLASTed against genome (e.g. oligonucleotides from *Arabidopsis thailiana* genome are selected, when a chip is built for the *homo sapien* DNA), (2) with known or predicted high hybridization affinity, and (3) with known or predicted low hybridization affinity.

This use case requires that databases of oligonucleotide libraries be accessed and an annotation analysis method be invoked to get annotation information for each oligonucleotide, if the required information is missing in the oligonucleotide library. The annotation information is used to select an appropriate set of oligonucleotides for the custom array design—for example, the oligonucleotides with GC content being 70% or up could be selected.

### Use case 3: annotation of a pre-built microarray for further analysis

This use case assumes a researcher has a pre-built microarray (e.g. Agilent’s 185K-CGI array, NimbleGen’s ENCODE array). To effectively normalize and analyze the experimental results, annotation information is needed for each oligonucleotide array on the microarray. For example, the locations and GC contents of oligonucleotides relative to those of genes of interest are very important in DMH experiments. So are the characteristics of nearby restriction enzyme sites, nucleotide polymorphisms, and simple repeats. We illustrate this use case through our DMH experiments on the Agilent’s 44K CpG island chip. Standard protocols are used to prepare DNA samples (Yan, Efferth et al. 2002). DNAs are digested with *Bfa*I, a 4-based restriction enzyme that cuts bulk genome into <0.10 kilobases, but retain GC-rich CpG island fragments. The methylation status of the assayed *Bfa*I fragments can be used to, for example, assess the methylation landscape of a gene’s promoter region. After linker ligation, digested DNAs are further restricted with methylation-sensitive restriction enzymes *Hpa*II and *Hinp*1I. Non-digested fragments are amplified by subsequent PCR, while unmethylated DNAs are digested away and cannot be amplified. The resulting amplicons are dye-coupled according to sample types (e.g. Cy3 and Cy5 for DNA from normal and cancer samples, respectively) and co-hybridized onto CpG island microarray. Intensive hybridization signals of oligonucleotides, which are mapped to DNA fragments flanked by *Bfa*I cleavage sites, are interpreted as a positive methylation event. Confidence in the methylation level of a fragment is statistically modeled using a combination of factors associated with the distribution of intensities, the number of *Hpa*II and *Hinp*1I recognition sites, and the length of *Bfa*I fragments. To develop hypotheses to explain these non-biologically related hybridization effects, structural characteristics concerning the oligonucleotides and *Bfa*I fragments can be collected, such as GC-content, Tm, known SNPs, and predicted secondary structure. To obtain such information, annotation operations should be invoked to calculate the needed structural characteristics for each oligonucleotide of interest. The annotation information can be input to normalization and data mining processes to better interpret the experiments.

Specifically, the appropriate annotation allowed for the development of a preprocessing method that modeled the different non-biological effects as a cubic-regression model and removed the estimated noise from the analyzed signal. The resulting preprocessed signal intensities could be clearly segregated into three classes interpreted as hypermethylated, hypomethylated, and undifferentiated. Previously, alternative methods had been employed for segregating the data into the three proposed states (Khalili, Potter et al. 2007). Though this method was relatively successful, it was hampered by the non-biologically relevant signal that lead to a higher than desired number of false classifications. By developing the signal-processing model on top of ArrayAnnotator-G, there is no need to redevelop the model for other chips (such as Agilents 185K- or 244K-CGI array) as would be necessary if the model were implemented in a closed system.

**Figure 1:**
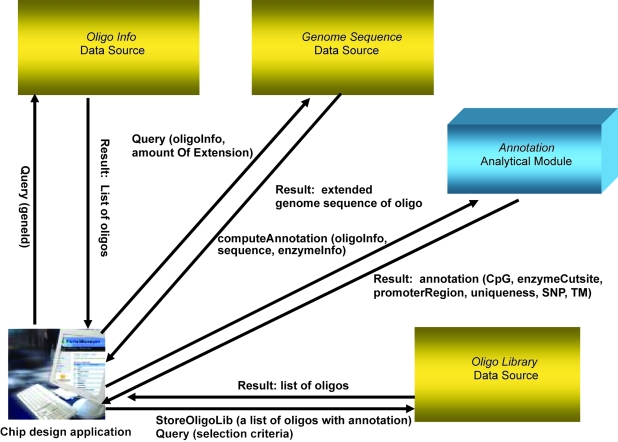
The design of the ArrayAnnotator-G: 1) three data source modules and one analytical module are presented and 2) an example set of APIs sent to each module and the output received from the module are shown along corresponding arrows between a chip design application client and each module.

Figure 1 illustrates the ArrayAnnotator-G framework to support these three usage scenarios. The framework consists of the following main modules—each of these modules can be used by itself or together depending on use case scenarios.

- *OligoInfo* data source: This data source provides basic information about oligonucleotides provided by a chip company (e.g. Agilent Technologies, Inc. provides information about more than 400,000 oligonucleotides that can be used in custom array design). This information includes chromosomal location and gene names. A client application can retrieve the list of oligonucleotides of interest and associated gene and chromosomal location information by submitting a query to the data source.
- *OligoLibrary* data source: This data source manages a set of oligonucleotides along with detailed annotation information. In contrast to the *OligoInfo* data source, this data source maintains more complicated annotations, which can be obtained through *Annotation* methods described below, as well as basic oligonucleotide information. This makes it possible for researchers to explore and select a set of oligonucleotides with their specific search criteria.
- *GenomeSequence* data source: This data source serves genome sequence information for a given oligonucleotide or any particular chromosomal location. In our current implementation, the *GenomeSequence* data service is implemented using a local Blat server from UCSC (http://genome.ucsc.edu/).
- *Annotation* analytical module: This module provides support for generating a list of annotations (see Table 1), which include the location of CpG Island close to a given oligonucleotide, the percentage of GC contents inside an oligonucleotide, the first exon location around a given oligonucleotide, the uniqueness of a given oligonucleotide, and restriction enzyme cleavage locations. To get the annotation information, a “compute annotation” request should be sent to the Annotation module with basic oligonucleotide information such as chromosomal location and its DNA sequence.

## 4. Implementation of ArrayAnnotator-G

ArrayAnnotator-G is designed as a service-based application and implemented using the caGrid infrastructure. Each data source in Figure 1 is implemented as a caGrid Data Service and the Annotation module is wrapped as a caGrid Analytical Service. The development process of a caGrid service from a data or analytical resource can be partitioned into two main steps. The first step involves the development of information models and object-oriented interfaces for a given resource. Any application which involves access to and management of data requires the design and implementation of one or more models to represent the information captured and referenced in the application. An information model expresses the data elements in the application, their attributes and definitions, and the relationships among the data elements so that the desired information can be searched and extracted correctly and efficiently. Information models and metadata are necessary to effectively manage, access, and share information. They also play crucial role in interoperability; if applications expose their data through well-defined information models, which are built on common data elements and controlled vocabularies, data can be accessed programmatically and interpreted correctly. In our application we employed an object-oriented approach, wherein data stored in the backend resources were exposed as objects in an information model through object-oriented interfaces. The second step implements Grid service interfaces and links these interfaces to the backend resource. The service developed in this step can also be configured to be a secure service. A secure service enforces authentication and authorization to control access to the service functionality.

### 4.1. Development of information models and caBIG silver level compliance

As we stated in Section 2, one of the important characteristics of the caBIG^TM^ program is the emphasis on syntactic and semantic interoperability. The program defines several requirements and processes for an application to be interoperable in the caBIG environment. These requirements are outlined in the caBIG compatibility guidelines^2^ developed by the caBIG community. Although an application can be implemented and deployed in the Grid environment without following these guidelines, this may hamper its interoperability with other caBIG applications in future. For this reason and to demonstrate the application of the various caBIG tools, we implemented the information models in the ArrayAnnotator-G application by following the processes developed and employed by the caBIG community.

The compatibility guidelines define several levels of maturity (Legacy, Bronze, Silver, and Gold) that quantify the degree of interoperability of a resource with other resources. The Silver level maturity indicates that the resource has object-oriented interfaces and serves objects, whose definitions are registered in the environment as common data elements and semantically annotated using controlled vocabularies. Silver level compliance is a required level of maturity for a resource before it can be exposed as a caGrid data or analytical service. The first step in caGrid service development is, therefore, to develop models for the data that will be served by a caGrid service and register them in the caBIG environment.

A suite of tools are available to facilitate development and registration of information models. These tools are built on the NCI Enterprise Vocabulary Services (EVS) and cancer Data Standards Repository (caDSR) (Covitz, Hartel et al. 2003; Phillips, Chilukuri et al. 2006), and the Mobius Global Model Exchange (GME) (Hastings, Langella et al. 2004) systems. The caDSR and EVS address the need for curation and management of common data types and controlled vocabularies used in cancer research applications. They are the authoritative sources of common data elements, vocabularies, and ontologies in caGrid. In this setting, data types used by a service are described in Uniform Modeling Language (UML) and converted into object class definitions, which are then registered in the caDSR. Proper semantic integration requires that each class and its attributes from a UML domain model be mapped to appropriate concepts in a controlled terminology. The concepts and definitions draw from vocabulary registered in the EVS, and their relationships are thus semantically described. In the Grid environment, XML is employed to represent registered data types, when instances of these data types are exchanged between clients and caGrid services, or between two caGrid services. XML schemas (XSDs) are used to represent the syntactic structure of each registered object. caGrid requires that XSDs for object models be published and managed in the Grid environment. Objects are serialized/de-serialized to/from XML documents that conform to published XML schemas, when they are transferred between two end points in the environment. The Mobius GME is the authoritative repository of the XML schemas that correspond to the object classes registered in caDSR. It is a DNS-like data definition registry and exchange service that enables services and clients to publish, retrieve, discover, and version XML schemas under namespaces. Namespaces allow creation of authoritative hierarchies on schemas and makes it easier to manage them. Hence, the first step for a service developer is to create data elements and object classes for the objects that will be exposed by the resource, and register them in the caDSR and EVS through a harmonization process. The second step is to create an XML schema for each object-type and register them in the Mobius GME under a namespace.

**Figure 2:**
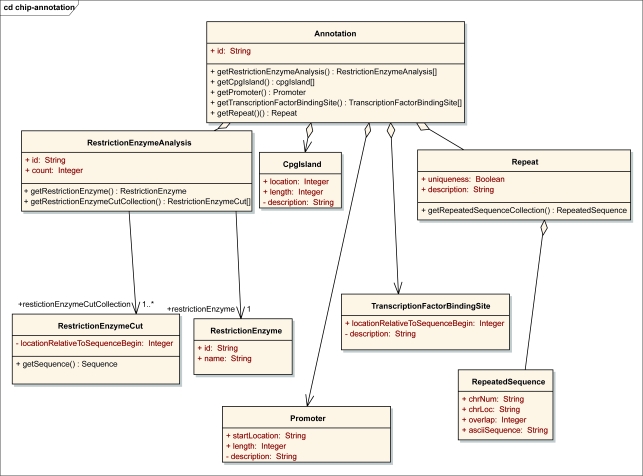
The UML model of the Annotation data type.

**Figure 3:**
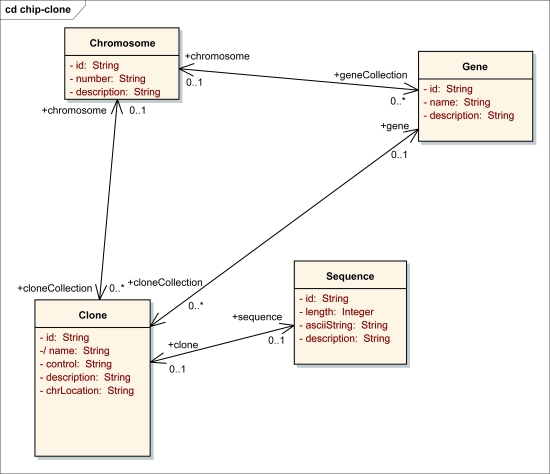
The UML model of the data types served by the OligoInfo and GenomeSequence data sources.

In the implementation of ArrayAnnotator-G, the information modeling and harmonization processes were carried out as follows. We used a UML modeling software^3^ to create the data elements in UML. We then annotated these UML models; documentation was created for each object class and definition for each attribute in the model. Using the UML modeling tool, a set of files containing the UML model, its representation in XML Metadata Interchange (XMI) format, and a human readable report were generated. The XMI file was further annotated using the Semantic Integration Workbench (SIW), which is a toolkit in the caCORE suite of tools(Covitz, Hartel et al. 2003; Phillips, Chilukuri et al. 2006). The SIW also generates an EVS report, which is reviewed by an EVS expert in the caDSR/ EVS team. All the files generated during this modeling process were submitted along with a UML submission form, which contains the name of our data model and the concept under which it should be placed, to the caDSR/EVS team for review and comments. The comments and modifications from the caDSR/EVS team were then incorporated into the model, and the model was resent for additional comments and approval. As examples, the UML models for the *Annotation* data type and data types served by the *OligoInfo* and *GenomeSequence* data sources are shown in Figure 2 and Figure 3. After the data elements had been harmonized and registered in the caDSR, we used the caCORE Software Development Kit (SDK) to develop corresponding object-oriented APIs to manage, query, and access the databases of objects of these data elements. We then created XML schemas for each of our data object classes registered in the caDSR and registered them in the Mobius GME under a namespace following the namespace conventions implemented in caGrid.

### 4.2. Implementation of data and analytical services

The next step in the implementation of ArrayAnnotator-G is to develop caGrid data and analytical services which expose data sources and analytical methods. A requirement of caGrid is that a service should have *strongly-typed* interfaces, which take objects as inputs and return objects as output and use XML for data exchange (i.e. objects are serialized into XML documents when they are exchanged between a client and the service or between two services). The method interfaces are *strongly-typed* in that the XML documents conform to XML schemas published in the environment. These schemas represent the structure of the objects published as common data elements. Service development also requires creation and coordination of a number of configuration files, source code files, metadata information, and directory structure so that the service can be deployed successfully. To make it easier for service developers to implement services, caGrid provides a toolkit called the Introduce toolkit (Hastings, Oster et al. 2007). Introduce automatically creates the runtime infrastructure needed to implement new analytic or data services. In essence, it is a program that automatically generates Java code. It makes it a simple matter to set up the basic “stub” classes and build environment for grid enabled services, easing the burden on the developer and allowing her to concentrate on the details of the domain specific implementation of his/her service. Introduce coordinates the process of specifying or discovering: 1) common data elements, 2) generating grid service code that makes use of the common data elements, 3) generation of service interface specifications, 4) XML serialization and de-serialization of objects and 5) specification of security constraints. In this section, we describe the use of the Introduce toolkit in implementing a service, called *OligoSelectService*, in our application.

The services shown in Figure 1 can be used to compute annotations for oligonucleotides. These annotations along with sequence information can be used to select a subset of oligonucleotides that are viewed as “good” for a given experiment or a microarray platform, or to be maintained as an oligonucleotide library (see the use cases in Section 3). One way to determine if an oligonucleotide is good or not, or to assign a goodness score to the oligonucleotide, is to compute the probe (oligonucleotide) target melting temperature (Tm) and the theoretical probe-target melting temperature (Tm2) through the secondary structure calculations and compare the temperature (Texp), at which the experiment will be run, to Tm and Tm2. For example, the best oligonucleotides can be marked as those oligonucleotides with Tm > Texp > Tm2, good ones with Tm > Texp and Tm > Tm2, and problematic ones with Tm ≤ Tm2 or Tm < Texp. The Annotation service in Figure 1 computes the Tm value as one of the oligonucleotide annotations. The OligoSelectService computes the Tm2 values and filters out the problematic oligonucleotides and assigns a score (“best”, “good”) to those selected. In our application, the computation of Tm2 values is done using a package called OligoArrayAux^4^ developed by N. Markham and M. Zuker at Rensselaer Polytechnic Institute. The package consists of a set of scripts and executables, which are called by these scripts. The OligoSelectService basically provides an interface to these scripts and executables and carries out the filtering and scoring of oligonucleotides. It consists of a selectOligos method, which takes an array of oligonucleotides, an array of related sequences, and an array of associated annotations as input and returns a list of selected oligonucleotides. The method transforms the sequence input into the format suitable for the OligoArrayAux package, call the scripts to compute Tm2 values for each sequence (hence, oligonucleotide), and select the subset of oligonucleotides that are scored as best or good and return them as output.

**Figure 4:**
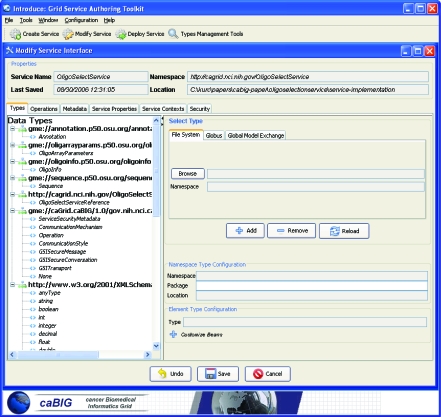
Introduce service creation and modification screen.

**Figure 5:**
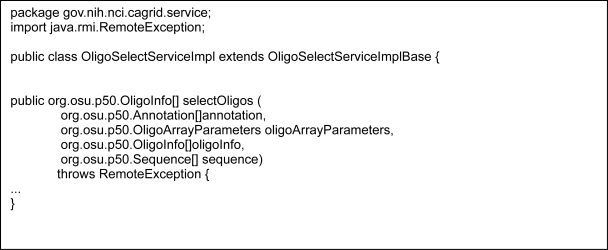
Oligo selection service interface in Java.

The first step in developing the service using the Introduce toolkit is to download the XML schemas that are registered in the Mobius GME for the input and output object types—Introduce obtains input and output object definitions from the GME; an XML schema can be obtained from the GME for each object type and schemas can be automatically downloaded and configured as method parameters. After the XML schemas have been downloaded, a service skeleton can be created using the Introduce Graphical User Interface (GUI) by specifying a name for the service (OligoSelectService in our case) and a directory in which all the service related files will be stored. The Introduce backend generates all of the basic source code, configuration files and the necessary build process for deployment of the service. The next step is to add a method to the service (selectOligos method in our example). The types of the input and output parameters (objects) of this method can then be selected from the list of the downloaded schemas. The basic service creation, schema download, and method creation screens are shown in Figure 4 for the OligoSelectService. Introduce will create the method interface definitions and associate them with the service. Figure 5 shows the method definition created in Java via the Introduce toolkit. All the code that is necessary to expose this method as a Grid interface, codes to do serialization and de-serialization of objects to and from XML documents, and the Grid service WSDL configuration files are automatically generated by the toolkit. Once the method is defined and created, the service developer should implement the backend of the method—in our case, this includes the code to transform objects into the suitable format for the scripts and executables in the OligoArrayAux package, pass the arguments to the executables, and parse the results and convert them into objects. In addition, the service developer needs to populate the metadata entries for the service (such as the name of the service, a description, which institution the service is hosted at) so that the service can be discovered based on this metadata. Introduce also allows a service developer to create and deploy a service as a secure service, with support for authentication and authorization.

### 4.3. Client programs

When a service implemented, a client API is also generated by the Introduce toolkit. Client APIs can be employed to develop client programs that can interact with services. Using client APIs generated for the services shown in Figure 1, we have developed a command-line client program for use case 2 presented in Section 3. The client program allows a researcher to build a custom array design by choosing a subset of oligonucleotides which satisfy various selection criteria—within a set of oligonucleotides, a screening process should be performed to select an appropriate subset of oligonucleotides for experiments. Such criteria can be given as flexible parameters so that researchers can adjust their protocols for custom array design. The client program can send a query with a gene name or gene id to the *OligoInfo* data service in order to retrieve a subset of oligonucleotides. Once the client program receives the output from the *OligoInfo* service, it sends another request to the *GenomeSequence* data service to retrieve a genomic nucleotide sequence for a given oligonucleotide with the specified amount of extension. After receiving the information, the client sends a request to the *Annotation* analytical service with the oligonucleotide information, the genome sequence information, and the restriction enzyme information. The Annotation service computes annotations for the oligonucleotide based on the sequence and enzyme information. This annotation information is used to select an appropriate set of oligonucleotides for the custom array design—for example, the oligonucleotides with GC content being 70% or up could be selected. The client program can request for a variety of annotations as shown in Table 1 and use a combination of annotation values for its own screening process. Once the client program has gathered all the needed information from the data and analytical services, the information can be stored in a local file so that it can be exported to another program such as Microsoft Excel for further manipulation. The rich annotation information obtained from the above process can also be stored in another data service, *OligoLibrary*, so that the annotated oligonucleotide information can be shared and easily accessed by researchers across different institutes and/or by collaborative peers within a single center.

**Figure 6:**
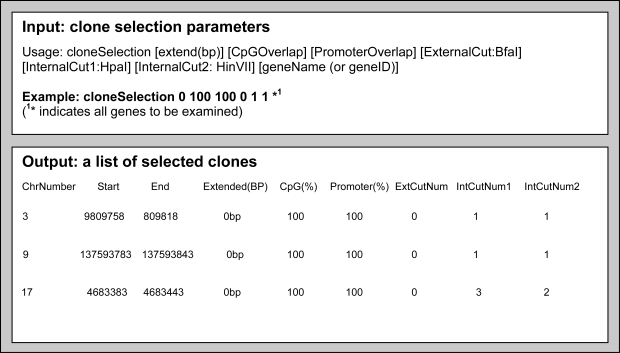
Example input and output of the command-line client program to select oligonucleotides using the ArrayAnnotator-G services.

An input/output example of this client program is shown in Figure 6. In this example, the user specifies 1) the amount of extended sequence around the oligonucleotide to examine, 2) the threshold value of CpG overlapping, 3) the threshold value of promoter overlap, 4) the minimum number of external enzyme cleavage locations, and 5) minimum number of internal restriction enzyme cleavage locations. In the example, the user uses 5 annotation values as oligonucleotide selection criteria: the percentage of overlapping with CpG island, the percentage of promoter region overlap, the number of external enzyme cleavage location (e.g. *Bfa1* enzyme), and the internal enzyme cleavage locations applying two Type IV enzymes, HipaI and HIPIII. As an example, the user sends a query for all oligonucleotides (specified as “*”), specifying threshold values for each selection criteria as 0, 100, 100, 0, 1. These threshold input parameters result in the set of oligonucleotides shown in the lower box in the Figure 6.

## 5. Conclusions

In this paper we reported on a novel application of the caBIG caGrid infrastructure in developing a Grid-enabled application framework to support the study of epigenetic alterations in cancer. Such studies require synthesis of information from multiple data sources and analysis modules to design experiments and analyze the experimental results. Our application framework provides support for annotation of oligonucleotides in custom array design. As an example, we have used ArrayAnnotator-G to select oligonucleotides for the CpG island microarray (CGpM). We selected a subset of the 60-mer oligonucleotides from the Agilent location analysis array (stored in the OligoInfo data service) that were within a CpG Islands (CGIs) promoter region. As a result, we got an array containing 40659 oligonucleotides designed from 12582 TSS within the promoter of 11351 unique genes. Other annotation information such as restriction enzyme cleavage and uniqueness information provided by the ArrayAnnotator-G application was used for normalization analysis.

The use of caGrid, a service-oriented and model-driven architecture, has enabled us to implement our software as modular, easily extensible, distributed framework. This implementation has several salient features. First, since it is service-based and all interactions with services are done through strongly-typed service interfaces, a back-end resource can be modified or a new data or analytical source can be easily added without requiring changes to the other modules and client applications. Second, the resources implemented as caGrid services can be deployed in a distributed environment, thus reducing dependency on a high-end centralized hardware and software platform. Third, since the services in our application are strongly-typed and use well-defined data types, collaborating researchers can interact with these services programmatically (i.e. they can develop client programs to access these resources) and parse the retrieved data correctly.

Our implementation demonstrates that service oriented architectures such as caGrid not only are applicable in designing and implementing applications for multi-institutional studies, but also are viable platforms to develop complex applications that carry out information synthesis and analytical computations on multiple data types and data sources. Moreover, the model-driven and strongly-typed nature of caGrid enables syntactic and semantic interoperability, thus facilitating the implementation of increasingly complex applications from autonomously developed, loosely coupled, potentially distributed components.

## Figures and Tables

**Table 1:** Annotations are important for experiment design and analysis of experimental results.

Annotation	Effect to experiments (or Analysis)
Promoter region	It has been demonstrated that the methylation of a gene’s promoter region effects the gene’s transcription.
CpG island	It is generally accepted that it takes a stretch of methylation changes to alter gene expression. This type of scenario occurs in CpG islands.
GC contents	Effective hybridization correlates with high GC content.
Gene	Focus on known/hypothetical/proposed genes of interest.
Restriction enzyme cleavage fragments (Type II enzymes)	When Type II restriction enzymes are used to generate fragments, the size and content of the generated fragment is critical to successful hybridization. Oligonucleotides with a cleavage site is in the middle of its sequence should not have a target in the sample (because the target should be cleaved by the enzyme) and therefore should not have signal in any experiment.
Methylation-sensitive enzyme cleavage sites (Type IV enzymes)	In DMH experiments, oligonucleotides that do not contain any methylation-sensitive cleavage sites are expected to have targets always present in the sample, and therefore have signal in every experiment.
Tm	The probe to target melting temperature (Tm) has been shown to be correlated with oligonucleotide (probe) hybridization levels. To reduce experimental error, the Tm values for the oligonucleotides should be homogeneous.
Uniqueness	If an oligonucleotide’s target is not unique (*i.e.* there are multiple regions in the DNA that may hybridize to the oligonucleotide), then the DNA region being assayed is not clear and the oligonucleotide must be discarded.
Repeat-masked region	The oligonucleotides within repeat-masked region would bias the experimental results.
Secondary structure	Secondary structures may inhibit target to oligonucleotide hybridization.

## Footnotes

1 The caGrid version 0.5, which was released to the caBIG community in September 2005, employed the OGSA standards, whereas the caGrid version 1.0, released in December 2006, is built on the WSRF standards.

2 https://cabig.nci.nih.gov/guidelines_documentation/caBIGCompat-GuideRev2_final.pdf

3 In our case we employed the Enterprise Architect commercial software from Sparx Systems, but there are a number of open-source UML modeling tools.

4 http://www.bioinfo.rpi.edu/applications/hybrid/OligoArrayAux.php
